# Clinical utility of the Glasgow Prognostic Score in patients undergoing curative nephrectomy for renal clear cell cancer: basis of new prognostic scoring systems

**DOI:** 10.1038/bjc.2011.556

**Published:** 2011-12-13

**Authors:** G W A Lamb, M Aitchison, S Ramsey, S L Housley, D C McMillan

**Affiliations:** 1 Department of Urology, Gartnavel General Hospital, Glasgow G12 0YN, UK; 2 Academic Unit of Surgery, University Department of Surgery, Royal Infirmary, Glasgow G31 2ER, UK

**Keywords:** renal cancer, nephrectomy, tumour stage, systemic inflammatory response, Glasgow Prognostic Score

## Abstract

**Background:**

Measurement of the systemic inflammatory response in malignancy has been recently refined using a selective combination of C-reactive protein and albumin (modified Glasgow Prognostic Score, mGPS). This has prognostic value in patients with metastatic kidney cancer. This study examines the prognostic value of the mGPS in patients undergoing curative nephrectomy for clear cell cancer.

**Methods:**

Patients with localised renal cell carcinoma undergoing potentially curative resection between March 1997 and July 2007 in a single institution were prospectively studied. The mGPS, University of California Los Angeles Integrated Staging System (UISS), ‘Stage Size Grade Necrosis’ (SSIGN), Kattan and Leibovich scores were constructed.

**Results:**

A total of 169 patients were studied. The minimum follow-up was 49 months; the median follow-up of the survivors was 98 months. During this period, 35 patients died of their cancer; a further 24 patients died of intercurrent disease. On univariate survival analysis of the scoring systems, Kattan (*P*<0.05), UISS (*P*<0.001), SSIGN (*P*<0.001) and Leibovich (*P*<0.001) were significantly associated with cancer-specific survival. Using cancer-specific mortality at 4 years as an endpoint, the area under the receiver operator curve was 0.726 (95% CI 0.629–0.822; *P*=0.001) for Kattan, 0.776 (95% CI 0.671–0.880; *P*<0.001) for UISS, 0.812 (95% CI 0.733–0.892; *P*<0.001) for SSIGN, 0.778 (95% CI 0.666–0.889; *P*<0.001) for Leibovich and 0.800 (95% CI 0.687–0.912; *P*<0.001) for the mGPS scoring system. On multivariate analysis of significant independent scoring systems and mGPS, UISS (HR 3.08, 95% CI 1.54–6.19, *P*=0.002) and mGPS (HR 5.13, 95% CI 2.89–9.11, *P*<0.001) were significant independent predictors of cancer-specific survival.

**Conclusions:**

The present prospective study shows that the mGPS, an inflammation-based prognostic score, is at least equivalent to and independent of other current validated prognostic scoring systems for patients undergoing curative nephrectomy for renal clear cell cancer. The mGPS is simple, measured preoperatively, based on well-standardised, widely available protein assays, and therefore provides an objective and rational basis before treatment for future staging systems in patients with operable renal cancer.

Renal cell cancer, although the twelfth most common cause of cancer death, is one of the most lethal urological cancers. Each year in the United Kingdom, there are ∼3500 new cases and ∼30% of these patients present with metastases. Overall survival is poor; even in those who undergo potentially curative resection, in high-risk groups, up to 50% may develop metastases within 2 years of surgery but only approximately half survive for 5 years (Cancerstats, www.cancerresearchuk.org).

The emergence of multikinase inhibitors has changed the landscape of therapy for patients with advanced disease significantly (Motzer *et al*, 2007). The role of these agents in adjuvant and neoadjuvant setting is not clear and is being addressed in ongoing clinical trials (SORCE, ASSURE). Recognition of patients at high risk of relapse is paramount both for follow-up and for consideration for therapeutic trials. To date, staging and stratification of patients with renal cancer is based predominantly on pathological criteria with other host factors, Kattan (Sorbellini *et al*, 2005), University of California Los Angeles Integrated Staging System (UISS) (Zisman *et al*, 2001) and Leibovich (Leibovich *et al*, 2003).

It is now recognised that disease progression in cancer patients is not only solely determined by the local characteristics of the tumour but also by the host inflammatory response (Colotta *et al*, 2009; Hanahan and Weinberg, 2011). In particular the systemic inflammatory response, as evidenced by inflammation-based prognostic scores, has an important role in the progression of a variety of common solid tumours, including renal cancer (Roxburgh and McMillan, 2010; Proctor *et al*, 2011a, 2011b).

The systemic inflammatory response, as evidenced by an elevated systemic C-reactive protein, has been shown to confer a negative prognostic outcome on patients undergoing curative nephrectomy for renal cell carcinoma (Karakiewicz *et al*, 2007; Ramsey *et al*, 2008; Lamb *et al*, 2008; Iimura *et al*, 2009). It has recently been suggested that C-reactive protein, specifically as a continuous variable, be incorporated into existing prognostic algorithms to maximise predictive ability (Johnson *et al*, 2010).

The measurement of the systemic inflammatory response has been recently refined using a selective combination of C-reactive protein and albumin (termed the Glasgow Prognostic Score, GPS). This has been shown to be a prognostic, independent of tumour stage, in a variety of gastrointestinal cancers (McMillan 2008, 2009; Proctor *et al*, 2011a). It also has been shown to have prognostic values in patients with metastatic kidney cancer (Ramsey *et al*, 2007).

The aim of the present study was to examine the prognostic value of the GPS in patients undergoing curative nephrectomy for clear cell cancer. Also, to examine how it compared with existing prognostic algorithms.

## Materials and methods

Patients with renal cell carcinoma, who, on the basis of surgical findings and preoperative computerised tomography of the chest and abdomen, underwent potentially curative resection between March 1997 and July 2007 in the North Glasgow NHS Trust were prospectively included in the study. Patients with nodal or distant disease on preoperative CT scanning were excluded from this study. Therefore, lymphadenectomy was not routinely performed. At this time, no patient showed clinical evidence of infection or other inflammatory conditions. No patient had nodal or metastatic disease, and all macroscopic tumours were removed at nephrectomy with subsequent negative surgical margins. Patients were staged pathologically according to the 1997 UICC TNM classification of renal tumours (Sobin and Wittekind, 1997). Tumours were graded according to criteria set out by Fuhrman *et al* (1982). Only clear cell cancers were included for analysis, all other histological subtypes being excluded. No patients were included in tyrosine kinase inhibitor trials, although patients developing metastases after 2008 were all given first-line Sunitinib, a multikinase inhibitor.

Clinical stage and performance status (Eastern Cooperative Oncology Group, ECOG-ps) were recorded before surgery. Routine laboratory measurements including C-reactive protein and albumin were performed preoperatively. The limit of detection of the assay was a C-reactive protein concentration lower than 6 mg l^−1^. The inter- and intra-assay variability of C-reactive protein and albumin were <5%. A C-reactive protein concentration of >10 mg l^−1^ was considered to indicate the presence of systemic inflammatory response (Roxburgh and McMillan, 2010).

Patients who survived to discharge were followed up according to Leibovich risk stratification protocol; patients classified as low risk underwent annual ultrasound scan and chest X-ray, intermediate risk underwent 6 monthly computed tomography scan for 2 years then annually until 5 years, and those classified as high risk underwent 6 monthly computed tomography scan for 3 years and then annually until 5 years. Thereafter, patients were followed up with annual ultrasound scan and chest X-ray.

The Research Ethics Committee of North Glasgow NHS Trust approved the study.

The UISS score was derived as previously described (Zisman *et al*, 2001). Briefly, tumour stage, Fuhrman grade and ECOG-ps are combined to stratify patients into low, intermediate or high risk.

The Stage Size Grade Necrosis (SSIGN) score was derived as previously described (Frank *et al*, 2002). Patients are awarded scores based on T stage, nodal disease, tumour size, nuclear grade, presence or absence of tumour necrosis, and the presence or absence of metastases. Patients with scores of 0–2 were classified as low risk, 3–5 as intermediate and 6 or greater as high risk.

The Kattan score was derived as previously described (Sorbellini *et al*, 2005). A nomogram is used to award points regarding presenting symptoms, tumour histology, tumour size and T stage to derive a total score, which can be used in the nomogram to estimate a 5-year recurrence free survival. Patients with total scores of <70 were classified as low risk, 70–100 as intermediate and >100 as high risk.

The Leibovich score was derived as previously described (Leibovich *et al*, 2003). Patients are awarded scores based on T stage, nodal disease, tumour size, histology, and the presence or absence of symptoms. Patients with scores of 0–2 were classified as low risk, 3–5 as intermediate and 6 or greater as high risk.

The modified Glasgow Prognostic Score (mGPS) was calculated as previously described (McMillan, 2008; Table 1); patients with an elevated C-reactive protein concentration (>10 mg l^−1^) and a decreased albumin concentration (<35 g l^−1^) was assigned score 2. Those patients with an elevated C-reactive protein concentration (>10 mg l^−1^) was assigned score 1 and patients with a C-reactive protein concentration of <10 mg l^−1^ and any albumin concentration was assigned score 0.

### Statistics

Grouping of the variables was carried out using standard thresholds. Inter-relationships between variables were assessed using contingency table analysis with the X^2^ test for trend as appropriate. Survival analysis was performed using the Cox's proportional hazards model. Deaths up to the end of July 2011 were included in the analysis. Multivariate survival analysis was performed using a stepwise backward procedure to derive a final model of the variables that had a significant independent relationship with survival. To remove a variable from the model, the corresponding *P*-value had to be >0.10. Analysis was performed using SPSS software (SPSS Inc., Chicago, IL, USA).

## Results

The relationship between clinicopathological factors and cancer-specific survival in patients undergoing curative nephrectomy for renal clear cell cancer is shown in Table 2. A total of 169 patients were studied. The majority was male (63%), under the age of 60 years (54%), had good performance status (82%), had T stage I/II disease (53%) and had absence of tumour necrosis (62%). The majority had an mGPS of 0 (69%) before surgery.

The minimum follow-up was 49 months; the median follow-up of the survivors was 98 months, no patient was lost to follow-up. During this period, 35 patients died of their cancer; a further 24 patients died of intercurrent disease. On univariate cancer-specific survival analysis, performance status (*P*<0.10), T stage (*P*<0.001), tumour size (*P*<0.10), grade (*P*<0.001), necrosis (*P*<0.01), C-reactive protein (*P*<0.001) and mGPS (*P*<0.001) were significantly associated with cancer-specific survival (Table 2). On multivariate analysis of these significant covariates, only grade (HR 1.72, 95% CI 1.15–2.56, *P*<0.01) and mGPS (HR 6.65, 95% CI 3.71–11.93, *P*<0.001) were significant independent predictors of cancer-specific survival.

On univariate overall survival analysis, performance status (*P*<0.05), T stage (*P*<0.001), tumour size (*P*<0.01), grade (*P*<0.001), necrosis (*P*<0.001), C-reactive protein (*P*<0.001) and mGPS (*P*<0.001) were significantly associated with overall survival (Table 3). On multivariate analysis of these significant covariates, only necrosis (HR 1.88, 95% CI 1.01–3.48, *P*<0.05) and mGPS (HR 4.17, 95% CI 2.48–7.03, *P*<0.001) were significant independent predictors of overall survival.

On univariate survival analysis of the scoring systems, Kattan (*P*<0.05), UISS (*P*<0.001), SSIGN (*P*<0.001) and Leibovich (*P*<0.001) were significantly associated with cancer-specific survival. Using cancer-specific mortality at 4 years as an endpoint, the area under the receiver operator curve was 0.726 (95% CI 0.629–0.822; *P*=0.001) for Kattan, 0.776 (95% CI 0.671–0.880; *P*<0.001) for UISS, 0.812 (95% CI 0.733–0.892; *P*<0.001) for SSIGN, 0.778 (95% CI 0.666–0.889; *P*<0.001) for Leibovich and 0.800 (95% CI 0.687–0.912; *P*<0.001) for the mGPS scoring system. On multivariate analysis of Kattan, UISS, SSIGN and Leibovich scoring systems, UISS (HR 3.78, 95% CI 1.91–7.50, *P*<0.001) and Leibovich (HR 2.36, 95% CI 1.40–3.96, *P*=0.001) were significant independent predictors of cancer-specific survival (Table 2). On multivariate analysis of these significant independent scoring systems and mGPS, UISS (HR 3.08, 95% CI 1.54–6.19, *P*=0.002) and mGPS (HR 5.13, 95% CI 2.89–9.11, *P*<0.001) were significant independent predictors of cancer-specific survival.

Univariate overall survival analysis of the scoring systems were significantly associated with overall survival (Table 3). On multivariate analysis of these significant covariates, UISS (HR 3.20, 95% CI 1.86–5.49, *P*<0.001) and Leibovich (HR 1.96, 95% CI 1.28–3.01, *P*<0.01) were significant independent predictors of overall survival. On multivariate analysis of these significant covariates and mGPS, UISS (HR 3.09, 95% CI 1.82–5.25, *P*<0.001) and mGPS (HR 3.38, 95% CI 2.17–5.24, *P*<0.001) were significant independent predictors of overall survival.

**Figure 1:**
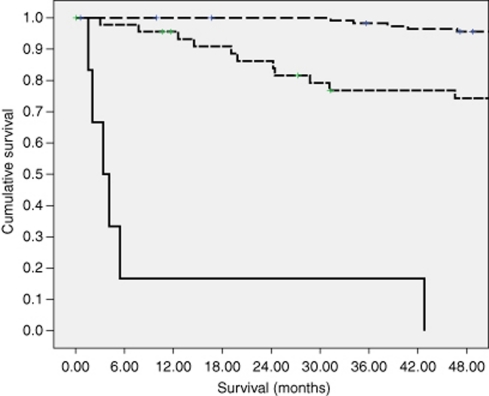
The relationship between the mGPS (0, 1 and 2 from top to bottom) and cancer-specific survival in patients undergoing curative nephrectomy for renal clear cell cancer.

The relationship between the mGPS and the clinicopathological characteristics are shown in Table 4. An elevated mGPS was associated with a greater proportion of patients with poorer performance status (*P*<0.05), advanced tumour stage (*P*<0.001), increased tumour size (*P*<0.001) and increased Fuhrman grade (*P*⩽0.001), presence of necrosis (*P*⩽0.001) and increased C-reactive protein concentration (*P*<0.001). An elevated mGPS was also associated with increased Kattan (*P*<0.001), UISS (*P*<0.001), SSIGN (*P*<0.001) and Leibovich (*P*<0.001) scores. Cancer-specific survival at 4 years for a mGPS of 0, 1 and 2 was 96%, 74% and 0%, respectively (*P*<0.001, Figure 1).

## Discussion

The present prospective study shows that the mGPS, an inflammation-based prognostic score, is at least equivalent to and independent of other current validated prognostic scoring systems for patients undergoing curative nephrectomy for renal clear cell cancer. The mGPS has much to commend it for routine clinical use in patients undergoing resection for renal clear cell cancer. It is simple, measured preoperatively, based on well-standardised, widely available protein assays and has been shown to have prognostic value in metastatic renal cancer (Ramsey *et al*, 2007) and in other resectable tumours (Roxburgh and McMillan, 2010). Therefore, the mGPS provides an objective and rational basis before treatment for future staging systems in patients with operable renal cancer.

Based on the present results, it would be important to examine whether a new prognostic scoring system based on the mGPS could be introduced. Indeed, Iimura *et al* (2009) recently proposed and validated the combination of TNM stage and C-reactive protein as a simplified prognostic assessment for patients undergoing nephrectomy for renal clear cell carcinoma. The results of the present study may suggest that the combination of mGPS and Fuhrman grade would be of interest. However, the magnitude of the hazard ratio associated with the mGPS (HR 6.65) was considerably greater than that of grade (HR 1.72) and an increasing mGPS was significantly and directly associated with increasing grade suggesting that the additional value of the post-operative Fuhrman grade is limited.

The present prospective study although it highlights the strong independent prognostic value of the mGPS in operable renal clear cell carcinoma has a modest sample size, from a single centre, and requires to be validated in another centre and ideally within the context of a randomised trial. However, preoperative assessment of the mGPS can be carried out routinely in most clinical centres and therefore validation studies can be readily carried out. Therefore, if the present work is validated in other centres, the mGPS should form the basis of future prognostic scoring systems for primary operable renal clear cell cancer. For example, in the present study, those patients with a mGPS of 2 had an extremely poor outcome (almost all had died from their disease within 6 months and therefore consideration could be given as to whether they had disseminated disease and would benefit from an operation).

In the present study of operable renal cancer only 4% of patients were classified as having a mGPS of 2 in contrast to 12% of patients with metastatic renal cancer (Ramsey *et al*, 2007). Therefore, it may be questioned whether the mGPS of 2 actually adds to the predictive accuracy in the operable cohort. However, there was a clinical and statistical difference in 4-year survival of mGPS1 compared with mGPS 2 (74% *vs* 0%, *P*<0.001) in the present operable cohort. These results taken together with the consistent clear prognostic value of a mGPS 2 in a variety of operable common solid tumours including renal cancer (Roxburgh and McMillan, 2010) would support its routine inclusion in clinical studies.

The basis of the equivalent or superior prognostic value of the simple mGPS compared with the other factors (Proctor *et al*, 2011b) and now scoring systems in patients undergoing curative resection for renal clear cell cancer is not clear. It may be related to its close direct relationship with circulating interleukin-6 and interleukin-10 concentrations (Ramsey *et al*, 2006). These cytokines probably reflect T-lymphocytic/macrophage activation (Lucey *et al*, 1996) and are pivotal in promoting a continuing Th2 cytokine response that may be of primary importance in promoting tumour progression in renal cancer (Lucey *et al*, 1996; Bromwich *et al*, 2003). Furthermore, there is now good evidence of the central importance of the systemic inflammatory response promoting progressive nutritional and functional decline in the cancer patient (McMillan, 2008). An elevated mGPS before surgery might be a useful therapeutic indicator and this warrants further clinical investigation.

Identifying patients at high risk of progression and death is imperative when considering patients for adjuvant or alternatively neoadjuvant trial selection, particularly with the novel new chemotherapeutic agents now available. It remains to be established what treatment should be offered to patients at high risk of progression and death, as curative resection for renal clear cell cancer was not achieved in those patients with mGPS 2. The mGPS has the advantage of identifying these patients preoperatively and may alter the decision for surgery, although the effect of the cytoreductive influence on survival cannot be established from our data. Indeed in the presence of metastatic disease, the mGPS has been shown to independently predict cancer-specific survival (Ramsey *et al*, 2007). What remains to be examined is the significance of preoperative mGPS when selecting patients for cytoreductive nephrectomy.

In summary, the preoperative mGPS appears to be superior to other established prognostic factors and scoring systems, in predicting cancer-specific and overall survival in patients undergoing potentially curative nephrectomy for renal clear cell cancer. This requires validation in other series with a view to integrating the mGPS into routine patient stratification for risk in patients undergoing treatment for renal cancer.

## Figures and Tables

**Table 1:** The mGPS algorithm (McMillan, 2008)

**Feature**	**Score**
CR P⩽10 mg l^−1^	0
	
CR P>10 mg l^−1^	1
	
CR P>10 mg l^−1^	
	
Albumin <35 g l^−1^	2

Abbreviation: mGPS=modified Glasgow Prognostic Score.

**Table 2:** The relationship between clinicopathological factors and cancer-specific survival in patients undergoing curative nephrectomy for renal clear cell cancer: univariate and multivariate survival analysis

		**Univariate survival analysis**		**Multivariate survival analysis**	
	**Patients (*n*=169)**	**Hazard ratio (95% CI)**	***P*-value**	**Hazard ratio (95% CI)**	***P*-value**
Age group (⩽60/>60 years)	91/78	0.95 (0.49–1.85)	0.875		
Sex (male/female)	107/62	0.90 (0.45–1.81)	0.766		
ECOG-ps (0/⩾1)	139/30	1.92 (0.90–4.11)	0.091		0.873
T stage (I/II/III/IV)	69/21/75/4	2.39 (1.51–3.79)	<0.001		0.116
Tumour size (cm)	169	1.01 (1.00–1.01)	0.069		0.781
Fuhrmann grade (1/2/3/4)	19/68/54/26	2.15 (1.44–3.21)	<0.001	1.72 (1.15–2.56)	0.008
Necrosis (absent/present)	104/65	2.67 (1.37–5.24)	0.004		0.674
C-reactive protein (mg l^−1^)	169	1.01 (1.01–1.0)	<0.001		0.394
mGPS (0, 1, 2)	117/46/6	7.27 (4.32–13.79)	<0.001	6.65 (3.71–11.93)	<0.001
Kattan Score (<70/70–100/>100)	60/51/58	1.54 (1.02–2.32)	0.040		0.343
UISS (low/intermediate/high)	39/113/17	5.27 (2.77–10.02)	<0.001	3.79 (1.91–7.50)	<0.001
SSIGN (0–2/3–5/⩾6)	55/67/46	2.78 (1.70–4.55)	<0.001		0.450
Leibovich (0–2/3–5/⩾6)	114/45/9	3.24 (2.02–5.19)	<0.001	2.36 (1.40–3.96)	0.001

Abbreviations: CI=confidence interval; ECOG-ps=Eastern Cooperative Oncology Group performance status; mGPS=modified Glasgow Prognostic Score; SSIGN=Stage Size Grade Necrosis; UISS=University of California Los Angeles Integrated Staging System.

**Table 3:** The relationship between clinicopathological factors and overall survival in patients undergoing curative nephrectomy for renal clear cell cancer: univariate and multivariate survival analysis

		**Univariate survival analysis**		**Multivariate survival analysis**	
	**Patients (*n*=169)**	**Hazard ratio (95% CI)**	***P*-value**	**Hazard ratio (95% CI)**	***P*-value**
Age group (⩽60/>60 years)	91/78	1.20 (0.68–2.13)	0.529		
Sex (male/female)	107/62	1.07 (0.59–1.92)	0.831		
ECOG-ps (0/⩾1)	139/30	2.12 (1.12–4.03)	0.021		0.312
T stage (I/II/III/IV)	69/21/75/4	1.97 (1.37–2.83)	<0.001		0.244
Tumour size (cm)	169	1.01 (1.00–1.01)	0.005		0.839
Fuhrmann grade (1/2/3/4)	19/68/54/26	1.82 (1.30–2.55)	<0.001		0.237
Necrosis (absent/present)	104/65	2.91 (1.62–5.22)	<0.001	1.88 (1.01–3.48)	0.045
C-reactive protein (mg l^−1^)	169	1.01 (1.01–1.01	<0.001		0.372
mGPS (0, 1, 2)	117/46/6	5.70 (3.49–9.33)	<0.001	4.17 (2.48–7.03)	<0.001
Kattan Score (<70/70–100/>100)	60/51/58	1.59 (1.11–2.27)	0.012	0.58 (0.35–0.96)	0.033
UISS (low/intermediate/high)	39/113/17	4.37 (2.51–7.60)	<0.001	2.32 (1.25–4.30)	0.008
SSIGN (0–2/3–5/⩾6)	55/67/46	3.06 (1.98–4.73)	<0.001	2.73 (1.62–4.60)	<0.001
Leibovich (0–2/3–5/⩾6)	114/45/9	3.33 (2.19–5.06)	<0.001		0.839
mGPS (0, 1, 2)	117/46/6	5.70 (3.49–9.33)	<0.001	4.59 (2.68–7.84)	<0.001

Abbreviations: CI=confidence interval; ECOG-ps=Eastern Cooperative Oncology Group performance status; mGPS=modified Glasgow Prognostic Score; SSIGN=Stage Size Grade Necrosis; UISS=University of California Los Angeles Integrated Staging System.

**Table 4:** The relationship between the mGPS and clinicopathological characteristics in patients undergoing curative nephrectomy for renal clear cell cancer

	**mGPS 0 (*n*=117)**	**mGPS 1 (*n*=46)**	**mGPS 2 (*n*=6)**	***P*-value**
Age group (⩽60/>60 years)	62/55	26/20	3/3	0.828
Sex (male/female)	77/40	27/19	3/3	0.277
ECOG-ps (0/⩾1)	100/17	37/9	2/4	0.013
T stage (I/II/III/IV)	58/17/42/0	11/4/31/0	0/0/2/4	<0.001
Tumour size (cm)	5.5 (1.0–22.0)	7.5 (1.0–27.5)	11.0 (9.0–28.0)	<0.001
Fuhrmann grade (1/2/3/4)	12/55/38/11	7/13/15/10	0/0/1/5	0.001
Necrosis (absent/present)	80/37	24/22	0/6	0.001
C-reactive protein (mg l^−1^)	<5 (<5–10)	25 (11–272)	132 (13–215)	<0.001
Kattan Score (<70/70–100/>100)	50/33/34	10/18/18	0/0/6	<0.001
UISS (low/intermediate/high)	32/78/7	7/35/4	0/0/6	<0.001
SSIGN (0–2/3–5/⩾6)	47/48/22	8/18/19	0/1/5	<0.001
Leibovich (0–2/3–5/⩾6)	90/25/2	24/17/4	0/3/3	<0.001

Abbreviations: ECOG-ps=Eastern Cooperative Oncology Group performance status; mGPS=modified Glasgow Prognostic Score; SSIGN=Stage Size Grade Necrosis; UISS=University of California Los Angeles Integrated Staging System.
